# Genomes of two siphophages infecting *Rhizobium leguminosarum*: B1VFA and V1VFA-S, exhibiting phage-plasmid-like features

**DOI:** 10.1128/mra.00706-25

**Published:** 2025-09-10

**Authors:** Damitha Gunathilake, Anupama P. Halmillawewa, Keith D. MacKenzie, Christopher K. Yost, Michael F. Hynes

**Affiliations:** 1 Biological Sciences, University of Calgary 2129, Calgary, Alberta, Canada; 2 Biology Department, University of Regina 6846, Regina, Saskatchewan, Canada; Loyola University Chicago, Chicago, Illinois, USA

**Keywords:** bacteriophages, prophage-plasmids, *Rhizobium*, whole genome sequence, rhizobiophages

## Abstract

We report the genome sequences of two *Rhizobium*-infecting siphophages, B1VFA and V1VFA-S, isolated from Canadian agricultural soils. Both encode genes associated with plasmid maintenance, including *parA*, *parB*, and *repC*, and share similar genome architecture. These phages expand the diversity of rhizobiophages and may represent a distinct siphophage lineage.

## ANNOUNCEMENT

To contribute to the poorly known diversity of rhizobiophages, we isolated and sequenced two phages from rhizosphere soils in Canada: B1VFA from bean field soil in British Columbia (49°03′54″N 123°49′52″W) and V1VFA-S from vetch rhizosphere soil from Ontario (43°59′33.9″N 77°31′46.8″W).

B1VFA was isolated using *R. leguminosarum* strain VF39SM (now reclassified as *R. johnstonii*) (1), while V1VFA-S was isolated using *R. leguminosarum* strain 336 (2). Briefly, 10 g of rhizosphere soil was suspended in 50 mL of SM buffer, shaken at room temperature for 1 h, centrifuged at 6,000 × *g* for 10 min, and filtered through a 0.22-µm membrane. Then, 1 mL of the filtrate was added to 10 mL of an exponentially growing host culture in Tryptone-Yeast extract broth and incubated overnight at 28°C with shaking. The culture was filtered again, serially diluted, and plated by the agar double-layer method. Single plaques were picked, each suspended in 500 μL of SM buffer, serially diluted and repurified. The phage morphologies were observed under transmission electron microscope (TEM) following the method described in reference (3).

Phage DNA was extracted using the Phage DNA Isolation Kit (Norgen Biotek, Catalog 46800). Libraries were prepared with the NEBNext Ultra II FS DNA Library Prep Kit (New England Biolabs, Cat. E7805S) and sequenced on the Illumina MiSeq platform generating 240 bp paired-end reads. Read quality was assessed using FastQC v0.12.1 (4). Bases with quality score lower than phred33 were trimmed using Trimmomatic v0.39 (5). B1VFA was assembled using Unicycler v0.4.5 (6) and Geneious R11 v11.1 (7) independently to yield the same assembly with a single contig. Only Unicycler assembly produced a complete single contig for V1VFA-S genome. Gene prediction and annotation were conducted using the RAST server (8). Functional annotation was further curated with BLASTp (9), and NCBI conserved domain database (10). Phage terminal analyses were conducted with PhageTerm (11) and curated with read mapping in Geneious using Bowtie2 v2.4.5 (12) plugin. All software was used with default parameters.

**Fig 1:**
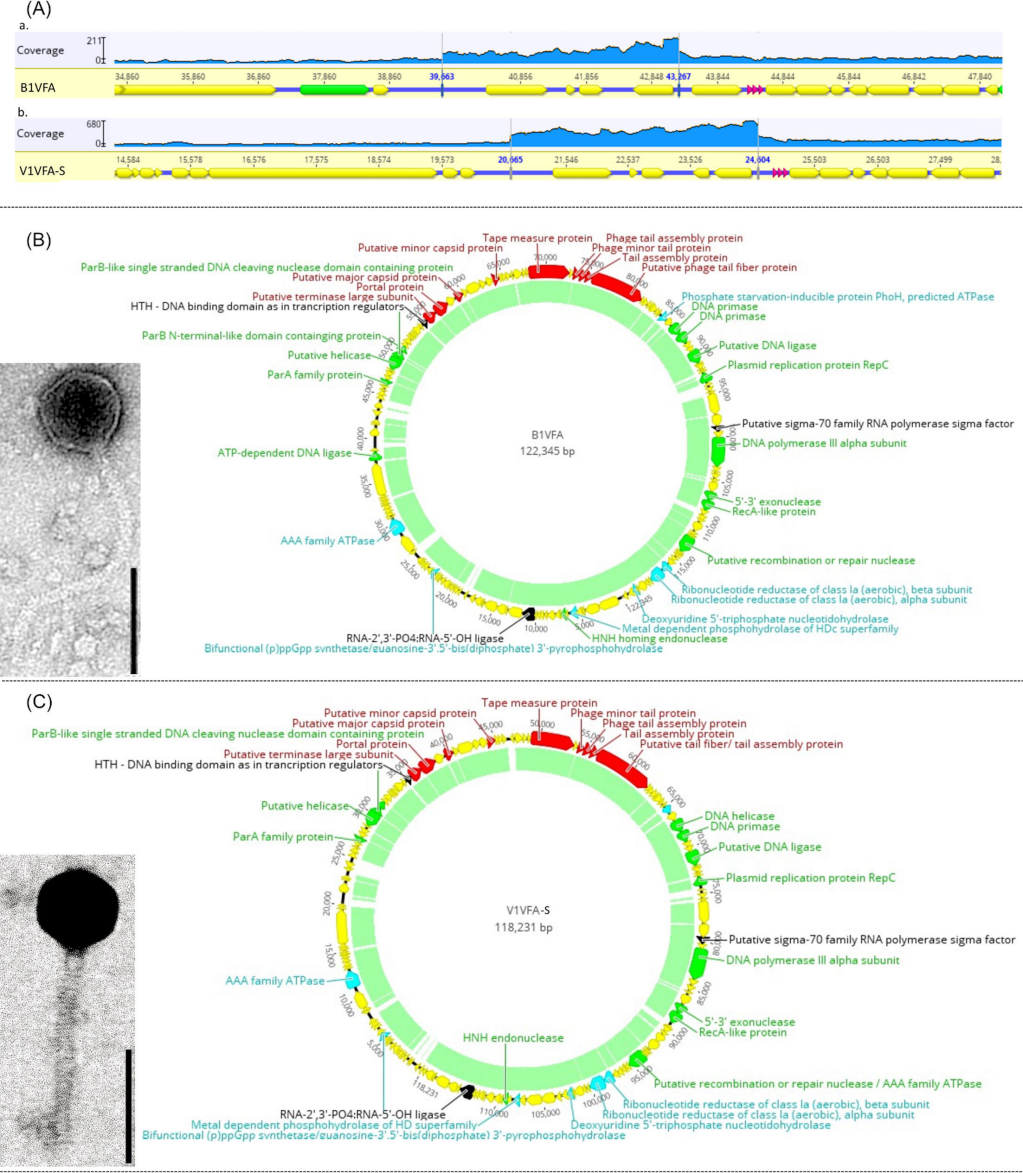
Genomic feature illustrations of B1VFA and V1VFA_S. (**A**) The high coverage regions of possible DTRs uncovered by read mapping in Geneious: a. B1VFA, b. V1VFA-S. (**B**) TEM image (scale bar shows 100 nm) and the circular genome organization of phage B1VFA depicted with overlapped DTRs. (**C**) TEM image (scale bar shows 100 nm) and the circular genome organization of phage V1VFA_S depicted with overlapped DTRs. Color scheme: red – structural, structure associated, and packaging proteins; bright green – replication, recombination, repair, and DNA modifying proteins; light blue – proteins involved in nucleotide metabolism; black – transcription related proteins; yellow – hypothetical and other proteins. CpG islands are indicated as light green blocks within the circle.

Both B1VFA and V1VFA-S have siphophage morphology with assembled genome sizes of 122,345 bp and 118,231 bp, respectively. All assembly and genome data are summarized in Table 1. Both phages have GC contents (Table 1) slightly lower than that of their host genome (61.2%) (13). In B1VFA, PhageTerm analysis indicated fixed termini with 3,605 bp long direct terminal repeats (DTR). In V1VFA-S, the PhageTerm predictions were inconclusive. However, read mapping showed elevated coverage in the terminal regions (Fig. 1), consistent with DTRs (3), in both phages.

**TABLE 1:** Genome features of B1VFA and V1VFA-S

Phage	Total no. of Illumina reads generated	Assembler	Average assembly coverage (×)	Assembled genome size with DTRs overlapping (bp)	Native genome size with separate DTRs (bp)	GC (%)	DTR size (bp)	No. of protein coding genes predicted by RAST	tRNAs predicted by RAST
B1VFA	21,332	Geneious and Unicycler	36.3	122,345	125,950	59.4	3,605	160	Cys-GCA, Met-CAT, Asn-GTT, pseudo-CCA
V1VFA-S	52,486	Unicycler	78.3	118,231	122,170	59.3	3,939	158	Met-CAT, Asn-GTT, pseudo-CCA

According to BLASTn, B1VFA shares 95.65% nucleotide identity (88% query cover) with V1VFA-S. Both phages have notable similarities only to a single plasmid (*R. beringeri* plasmid pRlX6, CP140813.1) and one other phage (*Rhizobium* phage RHph_X66, MW960030.1) in NCBI with 93%–94.5% identity (80%–88% query cover), suggesting these phages form a distinct lineage. Apart from the protein-coding genes (Fig. 1), these two phages code for 2–3 tRNA genes (Table 1). Interestingly, both genomes contain *parA*, *parB*, *repC*, and a protein with a predicted DNA-binding domain usually found in transcriptional regulators. This indicates that they are possible prophage-plasmids which can enter lysogeny by remaining and replicating as plasmids (14).

## Data Availability

The genome sequences of B1VFA and V1VFA-S are available in GenBank under accession numbers MT770738.1 and MT778838.1, respectively. Raw reads are available at the NCBI Sequence Read Archive (SRA) with accession numbers SRR34157448 and SRR34157516, respectively, under BioProject PRJNA1281619.
